# Correction: Mbigha Donfack et al. *Aedes* Mosquito Virome in Southwestern Cameroon: Lack of Core Virome, But a Very Rich and Diverse Virome in *Ae. africanus* Compared to Other *Aedes* Species. *Viruses* 2024, *16*, 1172

**DOI:** 10.3390/v17070939

**Published:** 2025-07-01

**Authors:** Karelle Celes Mbigha Donfack, Lander De Coninck, Stephen Mbigha Ghogomu, Jelle Matthijnssens

**Affiliations:** 1 Laboratory of Viral Metagenomics, Laboratory of Clinical and Epidemiological Virology, Department of Microbiology, Immunology and Transplantation, Rega Institute, KU Leuven, 3000 Leuven, Belgium; 2 Molecular and Cell Biology Laboratory, Biotechnology Unit, Department of Biochemistry and Molecular Biology, University of Buea, Buea P.O. Box 63, Cameroon

## Error in Figure 1 Position

In the original publication [1], there was a mistake in the figure position as published.

Figure position is in the Introduction section. The corrected figure position is in the Results section after Section 3.1 paragraph, which appears below. The authors state that the scientific conclusions are unaffected. This correction was approved by the Academic Editor. The original publication has also been updated.

Here is the corresponding text from the Results section where Figure 1 should be placed:

**3.**  **Results**

*3.1.*  *Each Sampling Site is Dominated by a Single Aedes Species*

This study focuses on analyzing the eukaryotic virome of the Aedes mosquito from four regions in southwestern Cameroon. In 2020, a total of 398 *Aedes* mosquitoes were captured from Bafoussam (*n* = 101), Edea (*n* = 96), Buea (*n* = 96) and Yaounde (*n* = 105) (Figure 1). Analyses of the mosquito distribution revealed that, *Ae. albopictus* species predominated in Edea, Buea, and Yaounde, while *Ae. africanus* was the most prevalent in Bafoussam. Small numbers of *Ae. simpsoni* were also captured, co-existing in regions predominated by *Ae. albopictus*. Only a single *Ae. aegypti* mosquito was captured in Edea (Figure 1).

**Figure 1:**
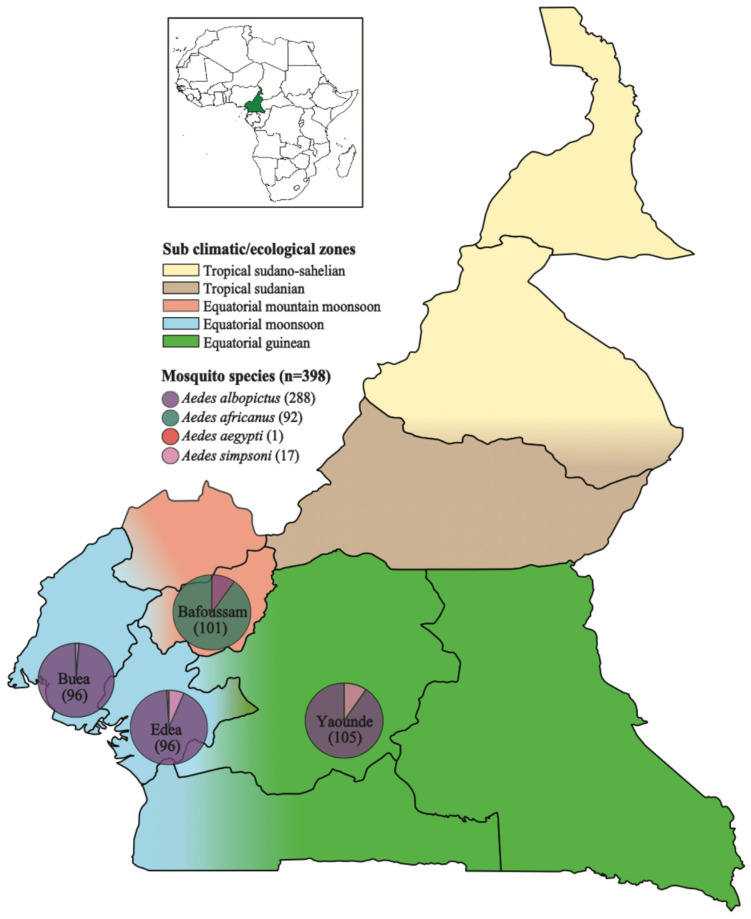
Map showing the climatic zones in Cameroon and the collection sites of *Aedes* mosquitoes in the southwestern part of Cameroon. The pie charts show the proportion of the different *Aedes* mosquito species found in each region.

## Error in Figure 1 Legend

In the original publication, there was a mistake in the figure legend as published.

Pink regions represent Equatorial monsoon zones and Blue regions represent Equatorial mountain monsoon zones. The corrected figure legend, Pink regions represent Equatorial mountain monsoon zones and blue regions represent Equatorial monsoon zones, appears above. The authors state that the scientific conclusions are unaffected. This correction was approved by the Academic Editor. The original publication has also been updated.

## Error in Figure 6 Text Language

In the original publication, there was a mistake in the text language in the figure as published. The text in the figure is in non-English. The corrected text language in the figure appears below. The text in the figure is in English. The authors state that the scientific conclusions are unaffected. This correction was approved by the Academic Editor. The original publication has also been updated.

**Figure 6:**
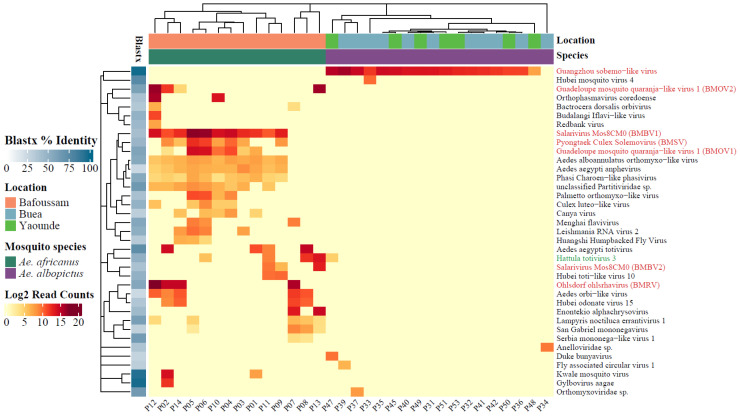
Read count of eukaryotic viral species on log2 scale. BLASTx percent identity to the most closely related reference sequence is shown in the shaded blue boxes. The virus name in green is the only virus species found in both *Aedes* species. Viruses in red were selected for qRT-PCR analysis, and the abbreviations of novel viruses with near-complete genomes (BLASTx < 90%) are shown within brackets.
